# Vertical Transmission of Hepatitis B Virus—An Update

**DOI:** 10.3390/microorganisms11051140

**Published:** 2023-04-27

**Authors:** Diana di Filippo Villa, Maria-Cristina Navas

**Affiliations:** Gastrohepatology Group, Faculty of Medicine, Universidad de Antioquia (UdeA), Calle 70 No. 52-21, Medellin 050010, Colombia; diana.difilippo@udea.edu.co

**Keywords:** hepatitis B virus (HBV), vertical transmission, risk factors, immunoprophylaxis, antiviral treatment, peripheral blood monocellular cells (PBMC)

## Abstract

Hepatitis B virus (HBV) infection is a major public health problem in the world. Approximately 296 million people are chronically infected. In endemic areas, vertical transmission is a common route of transmission. There are several strategies for the prevention of HBV vertical transmission, such as antiviral treatment during the third trimester of pregnancy and immunoprophylaxis to newborns that includes the administration of hepatitis B immune globulin (HBIG) and an HBV vaccine. Despite this, immunoprophylaxis failure can occur in up to 30% of infants born to HBeAg-positive mothers and/or with high viral load. Therefore, management and prevention of HBV vertical transmission is of paramount significance. In this article, we provided a review of the epidemiology, mechanisms of pathogenesis and risk factors of vertical transmission, as well as the strategies implemented to prevent the infection.

## 1. Introduction

The hepatitis B virus (HBV) is a major public health problem that affects approximately 2 billion people worldwide. Of these, 296 million are chronically infected [1]. In 2017, the World Health Organization set the goal to eliminate viral hepatitis by 2030, which is defined as 90% reduction in incidence and 65% reduction in liver-related deaths from the 2015 baseline [2].

HBV is a hepatotropic virus that belongs to the *Hepadnaviridae* family. The viral genome is a DNA of about 3.2 Kb that has four overlapping open reading frames (ORFs): S, X, P, and C. The S/preS ORF encodes the three form of hepatitis B surface antigen (HBsAg): long (HBsAg-L), medium (HBsAg-M) and small (HBsAg-S). The regulatory protein X (HBx) is transcribed from X ORF; the P ORF encodes the viral DNA polymerase; and the C ORF encodes the core protein (HBcAg) and a related protein called which is the precursor of the secreted protein known as antigen e (HBeAg) [3,4]. 

The diagnosis of HBV infection is established by detection of the molecular and serologic markers: viral DNA, HBsAg, HBeAg, anti-HBs, anti-HBe and anti-HBc (IgM and total antibodies) (Table 1).

Four routes of transmission have been described: (1) parenteral, i.e., by transfusion, re-used syringes and needles and contact of blood; (2) sexual; (3) vertical; (4) horizontal, i.e., by contact with body fluids, such as saliva, shared toothbrushes and secretory skin lesions [3]. 

In high endemic areas (Africa and Southeast Asia), HBV infection is acquired predominantly during the perinatal period or in early childhood [5,6]. Approximately 70–90% of infants born to HBeAg positive mothers in absence of active and passive immunization became chronically infected [6,7,8]. This risk is markedly reduced when HBV vaccine is given along with hepatitis B immune globulin (HBIG) in the first 12 h of life of the newborn along with antiviral therapy for the mother before delivery. Nevertheless, the immunoprophylaxis of some cases is not 100% protective; indeed, between 8% and 30% of infants can become infected [8,9,10,11]. Failures in immunoprophylaxis are related to the positivity of HBeAg and a high viral load in the mother, increasing the risk of vertical transmission.

## 2. Epidemiology of HBV Infection

According to the latest estimation, approximately two billion people worldwide have evidence of past or present HBV; 296 million individuals are chronically infected and almost 820,000 result in annual deaths from HBV-related liver disease [1,12]. The overall prevalence of HBsAg is 3.9% of the human population; however, it varies from country to country and depends on the complex interplay of behavioral, environmental, economic, geopolitical and host factors. 

**Figure 1:**
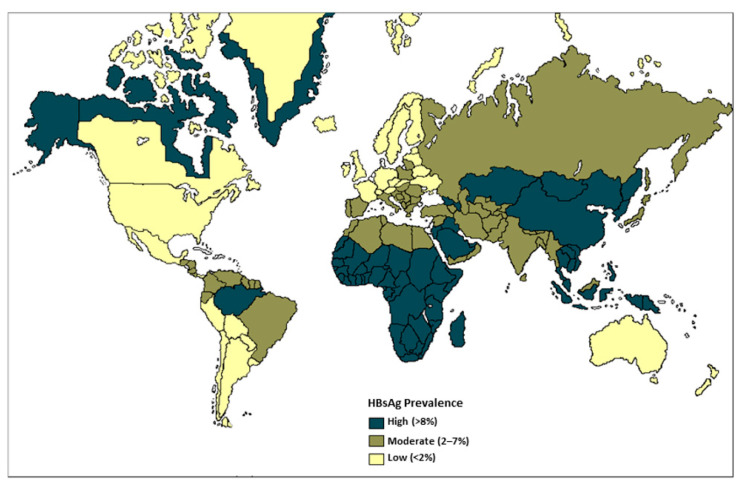
Global prevalence of chronic HBV. Adapted from source: Centers for Disease Control (CDC) 2020.

Three epidemiological patterns of HBV infection have been established according to the prevalence of HBsAg in general population: low endemicity (<2%; the United States of America (USA), Canada, Western Europe and some countries of South America), intermediate (2–7%; Middle East, Eastern Europe, Alaska, Greenland, Pacific Islands, Central America and South America) and high (≥8%; China, sub-Saharan Africa and the Amazon basin, among other regions) [6,12]. The variations in HBV endemicity are also frequently correlated with the predominant mode of transmission (Figure 1). 

In a highly endemic setting, most cases of hepatitis B infection are due to vertical transmission (mother-to-child infection) and horizontal transmission (exposure to infected family members) [13,14,15,16,17,18,19]. It has been estimated that 45% of the world’s population lives in endemic areas for HBV infection. In Asian and African countries, the vertical transmission is a common route as a considerable proportion of women are highly infectious at childbirth age, related to HBeAg positivity and a high viral load. Indeed, the prevalence of HBsAg among women of childbearing age in four provinces in China was higher than the national average prevalence for the same age group of women (11.8% vs. 6.6%). Moreover, HBsAg prevalence in women between 15 and 19 years of age was 5.21% (IC 4.05–6.36), and it was significantly higher (11.83%; IC 10.06–13.59) in women over the age of 40. This is probably due to the fact that older women were infected before the universal vaccination program [19]. Similar results were found in different regions of Africa, where the prevalence rank of HBsAg in pregnant women was 6.3–15.5% in western Africa, 7.7–9.2% in central Africa and 3.9–14.2% [18] in eastern and southern Africa. Given that the prevalence of HBsAg in pregnant women varies considerably from country to country, some studies suggest that the risk of perinatal infection may be lower in some African countries, and horizontal transmission would be the most frequent [15,17]. In any case, the prevalence of chronic HBV infection in women in these endemic regions represents a threat to maternal health and a risk of mother-to-child transmission. It demonstrates the conditions of vulnerability related to ethnicities, socio-economic status and education levels.

The Amazon basin is also a high endemic region for HBV infection [20,21,22,23,24,25]. The causes for high HBV prevalence are not completely understood, but it is likely a multifactorial problem: earlier age of pregnancy (relating to greater likelihood of high maternal viral load among women with CHB); crowded house; feeding small children with food previously chewed by the mothers; practices eliciting blood-to-blood contact, including ritual body modification or scarification; inadequate access to timely vaccination; and absence of effective primary health care and promotion and prevention programs [26,27]. A cross-sectional study was performed in 6 out of the 42 indigenous populations settled in the Peruvian Amazon. The prevalence of HBsAg in pregnant women was 2.11% (11/522) and the prevalence of anti-HBc was 42.06% (522/1241), indicating that a significant proportion of the population was exposed to the virus [24]. On other hand, the study showed that anti-HBc positivity in pregnant women was associated with age at pregnancy, first sexual intercourse at <16 years, bisexual partners and no condom use. 

A study in an indigenous population inhabiting the Curuçá and Itaquaí river basins of the Javari Valley in the Brazilian Amazon region described the markers of HBV infection. A total of 180 serum samples obtained from 87 (48.3%) men and 93 (51.7%) women were analyzed. The prevalence of HBV infection according to the ORF S detection (PCR) was 50.6% (44/87) in men and 51.6% (48/93) in women. Of these women, 21.5% (20/93) were pregnant and 75% (15/20) were HBV positive, showing a statistical difference (*p* = 0.009) when compared to the prevalence of HBV infection in non-pregnant women (40.4% (21/52)) [23]. The prevalence of HBV molecular markers found in pregnant women confirm the importance of prevention of vertical HBV transmission in the Amazon region´s populations. 

An interesting analysis of HBV infection was carried out in 1275 children and 572 mothers from 37 indigenous communities in the state of Amazonas, Colombia [25]. The average age of mothers and children was 32 ± 8.3 years (range 16 and 59 years) and 5 ± 3 years (range 6 months and 11 years old), respectively. The prevalence of anti-HBc in mothers’ population was 30.9% (176/572) and 9% (52/572) were positive for both anti-HBc and HBsAg markers. While 3.6% (46/1275) of children was positive for anti-HBc marker and 0.5% (7/1275) were also positive for HBsAgThe low prevalence of HBsAg observed in children confirmed the effectiveness of the hepatitis B vaccine program in this population; however, the frequency of HBV infection is still high in people born before 1994 when universal vaccination began, as reported in these indigenous mothers. This finding could be indicative of vertical transmission risk that could be increased in the absence of complete and timely immunoprophylaxis in the children. Indeed, this study demonstrated that being born to an HBsAg positive mother increases 2.5-fold the risk of the child being HBsAg + (OR = 2.45, 95% CI 1.33–4.46). Moreover, Garcia D et al. analyzed the vaccination records of this population and reported that the children with timely vaccination (birth dose and doses at 2, 4 and 6 months) had 70% less risk of being infected with HBV when compared with those who did not (OR = 0.23, 95% CI 0.09–0.51) [28]. 

A recently leading-edge study demonstrated important advances in achieving the goal of hepatitis B elimination in Colombia [29]. The study evaluated the feasibility of a two-phase approach to verify mother-to-child transmission of HBV in this country. In the first phase, municipalities with the highest risk of HBV transmission were identified, considering variables such as the number of HBV infections in pregnant women per 1000 individuals, coverage with third dose of pentavalent vaccine, the percentage of births occurring in health centers and seroprevalence data. The risk of hepatitis B was increased in municipalities with hepatitis B immunization low coverage and/or <90% of births in health centers The second part of the analysis included a serological household survey of children aged 5 to 10 years in the 36 municipalities with highest risk of HBV infection. A total of 3203 children from these municipalities were screened for HBV infection marker. HBsAg was not detected in any of the 3203 samples, yielding an upper 90% confidence limit of <0.1% prevalence [29]. Importantly, the study reported that there are still populations in which vaccination coverage is low, such as home-born children and indigenous communities. This result suggests that cultural traditions and/or barriers to access the health system may contribute to the higher prevalence of hepatitis B reported in these communities. Overall, the results of this study could indicate that Colombia is possibly on the right track to eliminating mother-to-child transmission of HBV; however, there is a need to strengthen screening during pregnancy, provide access to timely vaccination, establish systems to monitor HBV infection among pregnant women and complete follow-up testing of infants born to HBsAg-positive women. 

In regions of intermediate endemicity, such as Western Europe, most cases of HBV infection are acquired through sexual intercourse. In Italy, Russia and Turkey, the prevalence of chronic hepatitis B ranges from 3% to 10%, and unsafe injections are a major route of HBV transmission [30,31]. Interestingly, HBsAg prevalence in pregnant women of western and southwestern Europe countries was 0.1–4.4% [32], 1.18–4.3% in Iran [33] and 0.2–3.8% in some countries of South America (Peru, Venezuela, Brazil, Argentina and Colombia). This is in agreement with the low frequency of vertical transmission in these regions [28,34,35,36]. On the other hand, injection drug users are the most infected population in regions with low overall prevalence such as Eastern Europe and North America. Other populations at increased risk if HBV infection are prisoners, men who have sex with men, sex workers and homeless [37]. Studies carried out in pregnant women reported HBsAg prevalence from 0.3% to 2.4% [38,39] in the USA, from 0.76% to 0.85% in Australia [40] and from 1.4 to 1.5% in Japan [41]. Therefore, vertical transmission in these regions does not seem relevant.

## 3. Discovery of HBV Vertical Transmission

Saint-Vel’s 1862 report of severe hepatitis in pregnant women in Martinique laid the initial groundwork for studying the effects of HBV infection in pregnancy [42]. However, it was not until 1954 that the possibility of vertical transmission of HBV was considered based on a report of infants who developed hepatitis during the first two months of life and the evidence that some mothers were “silent carriers” of HBV; this was relevant to the problem of maternal transmission of this infection [43]. Later, a prospective study conducted in the USA and Europe revealed that in two-thirds of the infants of mothers who had acute hepatitis B at the end of pregnancy or after delivery became infected within one to six months of birth. In addition, the HBsAg was detected less frequently in newborns if maternal hepatitis occurs in the first trimester; therefore, transmission is less frequent when the mother is asymptomatic [44].

In a study carried out in newborns, three interrelated factors were found to increase the risk of HBV vertical transmission: a high titer of the HBsAg in the maternal circulation, presence of this marker in the umbilical cord blood and HBsAg+ in siblings. It was suggested that if babies remain antigenemic until adulthood, vertical transmission could account for an important proportion of asymptomatic cases (carriers) in areas of HBV high prevalence [45]. On the other hand, Okada et al. [46] described the HBeAg as an important factor to vertical transmission. In serum samples from 23 HBsAg-positive pregnant women and their babies, the HBeAg was detected in 43.4% (10/23) of women and the anti-HBeAg in 30.4% (7/23). All ten babies born to mothers HBeAg positive acquired persistent HBsAg, and all 10 siblings of these newborns were HBsAg+ and asymptomatic. In contrast, seven newborns of anti-HBe positive mothers were HBsAg negative, and none of their three siblings were positive for HBsAg. Furthermore, HBsAg was not detected in the serum of any of the fathers [46]. Based on these results, it can be concluded that HBeAg and anti-HBe may be used as indicators of HBV vertical transmission risk.

Although HBV transmission from infected mothers to their infants has been well documented in several studies, it is believed that in utero infection does not occur and the babies become infected by contact with maternal blood/fluids during delivery since serological signs of infection do not become evident until six weeks of age. To answer the question of whether a vertical transmission was in utero, an animal model of duck hepatitis B virus (DHBV) infection, a member of the *Hepadnaviridae* family and closely related to the HBV, was used [47]. The DHBV DNA was detected in 30/219 in both serum and liver embryos of infected Pekin ducks, and viral replication intermediates were identified in the liver on the 12th day of embryonic life. These findings were confirmed in another infection model, the woodchuck hepatitis virus (WHBV), another virus-related HBV. The WHBV DNA was detectable in the liver of 18/24 fetuses of infected woodchucks. Viral replicative DNA and supercoiled DNA, as well as WHBV surface antigen, were demonstrated in livers of two fetuses from late gestation (fetuses 5.5–7.0 cm in length and well-differentiated hepatocytes) [48]. Although maternal transmission to offspring may be different in humans, these observations in animal models were a first approach to understand HBV vertical transmission.

## 4. Vertical Transmission of HBV

The transmission of HBV infection from mother to child during pregnancy, delivery and/or breastfeeding is known as vertical transmission [1]. For HBV, vertical transmission is defined as positivity for HBsAg or HBV-DNA in infants at 6–12 months of life [6,49]. The presence of both HBsAg and viral DNA in serum at birth are often transient and do not always indicate newborn infection. The diagnosis of HBV infection is confirmed 6 months after birth, and if serological and molecular markers are still positive in a 1-year old baby, they indicate a chronic HBV infection. 

**Figure 2:**
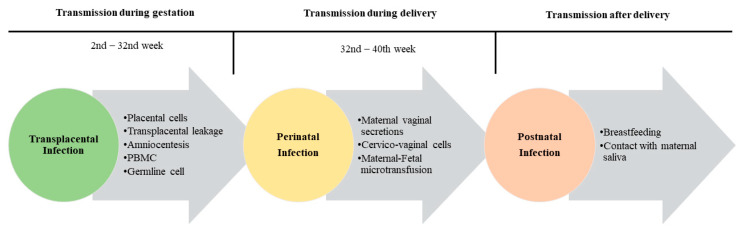
HBV vertical transmission mechanisms.

There are three possible routes for vertical transmission of HBV: (1) transplacental or intrauterine; (2) perinatal during delivery; (3) postnatal during care; (4) and/or through breast milk (Figure 2).

### 4.1. Transplacental Transmission

Transplacental transmission occurs in a minority of the cases of vertical transmission, and the mechanisms are not fully elucidated yet. There are some hypotheses of the possibility of transplacental transmission: the HBV can cross the placental barrier infecting placental cells; a transplacental leakage of maternal blood; during amniocentesis; through infected maternal peripheral blood monocellular cells (PBMC); or through germline cell infection (Figure 2).

**Figure 3:**
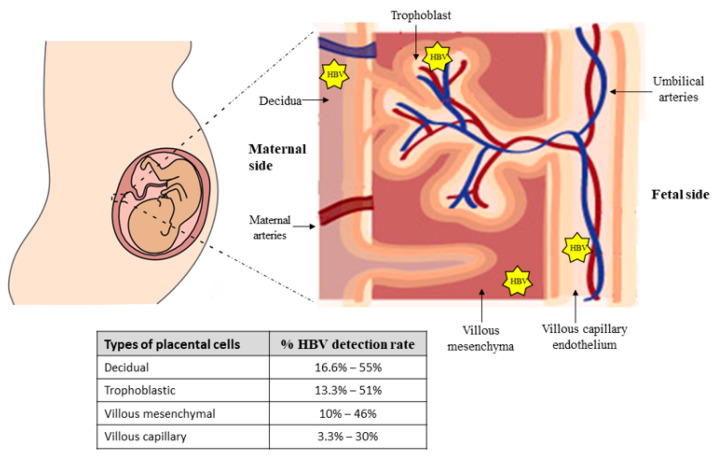
Representation of HBV-infected placental cells. While the percentage of HBV-positive cells decreases per layer when the virus passes from mother to fetus, there is a higher risk of vertical transmission upon contact with the endothelial cells of the villi when compared to the outermost placental layers.

Some studies have shown the ability of the virus to translocate through the placenta to the fetal trophoblast [10,50,51]. Thus, Chen et al. examined viral infection in 157 placental tissues obtained from infected pregnant women by detection of HBV DNA with PCR and in situ hybridization techniques and detection of HBsAg and HBcAg by immunohistochemistry assay. The HBV infection rate was higher in decidua cells (55.4%; 87/157) and in trophoblastic cells (51.0%; 80/157); compared with villous mesenchymal cells (46.5%; 73/157) and villous capillary endothelial cells (29.9%; 47/157) (*p* < 0.01) [10]. Similar results were reported by Xu et al [50]. In this study, the HBV infection rates on the 101 placenta samples (HBsAg, HBcAg or viral DNA) were 40.6% in trophoblastic cells, 36.6% in villous mesenchymal cells (37/101) and 18.8% in villous capillary endothelial cells. Both studies demonstrated that the HBV infection rate gradually decreases in the maternal-to-fetal placental layer. Therefore, HBV could cross the placental barrier, thus infecting and replicating in all types of placental cells before it reaches the fetus (Figure 3).

Another study determined the time when transplacental HBV infection occurs [51]. The HBV infection rates of 131 placentas from first trimester, second trimester and third trimester were 4.2% (1/24), 16.7% (1/6) and 44.6% (45/101), respectively. Additionally, the risk of HBV infection was 18.46 in villous capillary endothelial cells. These results demonstrate although the HBV infection may occur since week 19, it is more likely during the third trimester of pregnancy [51]. Further investigations are necessary to describe and understand the cellular and virological mechanisms that allow transplacental HBV infection in mother-to-child transmission cases.

HBV can also be transmitted to the fetus by leakage of maternal blood through the placenta into the fetal circulation. Probably during labor some placental cells are detached as a result of uterine contractions and maternal blood enters the fetal circulation, allowing viral particles from mother to establish infection in the fetus, especially in HBeAg-positive mothers and high viral load cases [52,53]. 

Amniocentesis is another possible mechanism of HBV transplacental transmission. This procedure may cause uterine or placental bleeding and may lead to blood exchange between mother and fetus [54]. Amniocentesis can expose the fetus to HBV via two routes: maternal-fetal blood exchange or ingestion of HBV-contaminated amniotic fluid. When the needle crosses the abdominal and uterine wall, it causes injuries to the chorionic villi and results in admixture of maternal and fetal blood. Additionally, uterine wall puncture also causes fetal–maternal bleeding through capillaries in the fetal membranes. By any of the routes, the fetus may enter in contact with virions due to the introduction of maternal infected blood into the fetal circulation or into the amniotic fluid. However, the published studies have not shown that amniocentesis significantly increases the risk of mother to infant transmission [55,56].

Viral particles have also been identified to circulate in maternal PBMC [57,58]. In a study carried out in 30 HBsAg-positive pregnant women and their aborted fetuses, the role of PBMC cell transportation from mother to baby in HBV intrauterine infection was investigated [57]. In the fetuses, HBV intrauterine infection rate was 43.3% (13/30). Moreover, the HBsAg was detected in 10% (3/30)of peripheral blood, 23.3% (7/30) of serum and 33.3% (10/30) of PBMC samples. Maternal–fetal PBMC transport was positively correlated with fetal PBMC HBV-DNA (*p* = 0.004). This study suggested that HBV intrauterine infection was primarily due to PBMC maternal–fetal transportation. This hypothesis was confirmed by a recent case-control study that evaluated HBV serologic markers in neonates of 312 HBsAg-positive mothers. Polymorphic markers (glutathione S transferase M1 -GSTM1- and angiotensin converting enzyme -ACE-) were used to detect trafficking of PBMCs from mother to child [58]. This study showed that 45.5% (142/312) of neonates were HBV infected in utero. Of the mother–baby pairs with information of polymorphic marker, 63.0% (75/119) showed mother-to-fetus PBMC transfer; of these, 76% (57/75) of the infants were HBV-DNA positive in PBMC. In comparison, only 25.0% (11/44) of the infants without mother-to-fetus PBMC transfer were HBV infected. These data demonstrated that mother-to-infant PBMC traffic increase the risk of HBV infection in newborn infants 9.5-fold (OR, 9.5; 95% CI, 3.71–24.91; *p* < 0.001) and therefore PBMC infection is an important risk factor for HBV vertical transmission [58]. 

Another important aspect to evaluate HBV infection in PBMCs is whether the presence of specific viral variants in this cell population can modulate the risk of mother-to-child transmission. In this context, a study analyzed HBV replication status in PBMC and pre-core/basal core promotor (PC/BCP) mutants in blood samples obtained from pregnant women [59]. In total, 37 women with chronic hepatitis B were included in the study; from these, eight had started NA therapy in the third trimester of pregnancy although all patients’ samples were collected before starting NA therapy. All infants received immunoprophylaxis and were HBsAg negative at 9–12 months of age. HBV-DNA was detected in 55% of PBMC from pregnant and/or postpartum women. The viral pregenomic RNA (pgRNA) was detected in 44% and the viral covalently closed circular DNA(cccDNA in 51% of the PBMC-tested. BCP mutants (A1762T, G1764A) were identified in 36% and PC mutants (G1896A) in 4% of cases [59]. On the other hand, the detection of HBV-DNA and/or variants in PBMC was not associated with HBeAg status, HBV-DNA, genotype, pregnancy–postpartum status or risk of liver disease during the median 4-year follow-up; however, the limited size of the study must be taken into consideration. These data suggest that HBV PC/BCP mutations are not related to mother-to-child transmission risk in cases of infants with complete and timely immunoprophylaxis. However, it should be taken into consideration that mothers with viral loads above 200,000 IU/mL in the third trimester received NA therapy, which significantly reduced the risk of in utero and at birth transmission. All these studies suggest a role of PBMC in HBV mother-to-child transmission. Additional research is necessary to understand if maternal PBMC HBV infection is productive and therefore generates virions that allows the subsequent newborn liver infection or if the PBMC infection modulated cellular mechanisms of immunotolerance and apoptosis that allow the HBV persistence in neonate.

The possibility of HBV intrauterine transmission via oocyte or sperm has been raised considering [49,60,61] that HBV DNA, pgRNA and HBsAg has been detected in oocytes and embryos from HBsAg-positive women during in vitro fertilization [61]. In addition, fluorescence in situ hybridization (FISH) revealed the presence of HBV DNA in oocytes and embryos from couples with at least one positive HBsAg [62]. Another study reported the HBV infection of infants of two cases of HBsAg positive oocytes [49]. In contrast, Jin et al. reported that babies born from in vitro fertilization using HBV positive oocytes and/or embryos were not HBV infected [63]. It is important to note that the fertilization technique had some impact on the viability of the virus in infected oocytes.

Using molecular hybridization and restriction enzyme patterns, the viral genome and integrated sequences were demonstrated in sperm samples obtained from 17 Hepatitis B patients (9 cases of acute hepatitis B and 8 cases of chronic infection) [64]. Moreover, the HBV DNA was detected in seminal fluid obtained from 319 patients with acute hepatitis, and integrated viral sequences was detected in 2 spermatozoa samples; however, the viral DNA was not detected in serum samples of these patients at the time of sperm collection. In addition, no HBV DNA was detected in semen samples obtained from chronic infection cases. 

Qun Xi et al. evaluated the hypothesis of vertical transmission via the germ line [65]. Sperm samples were obtained from 8 cases of chronic infection and three of them were HBV-DNA positive. However, none of their infants (born to HBsAg negative mothers) were HBV infected. These results suggested that HBV transmission to the fetus via the male germline is unlikely. According to the authors, it could be related to the presence of low number of viral particles in sperm could be rapidly eliminated by the maternal immune system and therefore prevent the virus transmission; it addition, the presence of protective antibodies (anti-HBs) was demonstrated in serum and uterine tissue samples obtained from 90% of wives of HBV-carrier husbands [65].Furthermore additional studies are required to understand the role of HBV infection in germ lines in intrauterine transmission.

### 4.2. Perinatal Transmission

Perinatal transmission is the most frequent route (30–40%) of vertical transmission. This mechanism is defined as infant contagion at the time of birth as a result of exposure to maternal vaginal secretions, epithelium cells and micro transfusion of maternal and fetal blood [66]. 

The presence of HBsAg in cord blood has been significantly associated with the length of labor when the first stage was more than nine hours (*p* < 0.03); a stronger association was found when labor lasted more than 11 h (*p* = 0.01) [66]. It has been reported that a high viral load (10^8^ UI/mL) in maternal blood entering a fetal body can result in fetal infection [67]; additionally, the HBsAg was detected in 96% of the vaginal fluid obtained from mothers, 2% of the breast milk samples and 90% of gastric aspirates from the babies. On the other hand, presence of HBsAg has also been demonstrated in 55–98% of vaginal epithelial cells and 12.1% of the cervico-vaginal cells along with detectable HBV DNA [68]. Consequently, the direct contact with infected cells or vaginal secretions when crossing the birth canal is the main mechanism for HBV transmission to infants.

Another topic that has been considered is the delivery mode and perinatal transmission [69,70]. In a study of 447 infants born to HBeAg- and HBsAg-positive mothers, a higher rate of infection was found in newborn delivered vaginally (24.9%, 96/385) than in delivered by caesarean (<10%, 6/62) [69]. However, no significant differences were observed in the positivity of HBsAg or anti-HBsAg among three groups of babies born to infected mothers, 144 delivered vaginally, 40 by obstetric forceps or vacuum extraction and 117 by cesarean at follow-up periods (1, 4, 7 and 12 months of age) [70]. These findings suggest that failures in immunoprophylaxis (incomplete vaccination schemes or the non-administration of HBIG, as evidenced in this study) are the cause of HBV transmission in babies independent of the delivery mode.

### 4.3. Postnatal Transmission 

The HBV transmission in the postpartum period represents a less common (<7%) way of vertical transmission; however, there are some reports of high proportion (34%) of infection in postnatal period [71]. Some mechanisms have been suggested for HBV postpartum transmission related to infant contact with HBV-contaminated maternal secretions such as breastfeeding, ingestion of food previously chewed by the mother, maternal kissing in the mouths of infants and lack of effective hand hygiene among healthcare personnel involved in the postpartum care of mothers and infants (Figure 2) [72,73,74,75,76].

In the early 1970s, some researchers published data on HBsAg transmission in breast milk of chronically infected mothers [73,74]. Although HBsAg, HBeAg and viral DNA have been detected in the colostrum and the breast milk, there is no evidence that breastfeeding increases the risk of mother to child transmission of HBV, as described by Chen X. et al., where HBsAg prevalence in breastfed and formula-fed children was 1.5% and 4.7%, respectively (*p* = 0.063) [72]. These results indicate that the risk of transmission associated with breast milk is small when compared to the risk of infection in the infant due to contact with maternal blood or fluids during the delivery.

However, most of studies have neither quantified maternal viral load nor explored the correlation between the rate of vertical transmission and the duration of breastfeeding. On the other hand, experts have suggested that lesions in the breast tissue, such as cracked or bleeding nipples or lesions with serous exudates, could be an important source of infectious viral particles for the infant; however, this issue has not yet been studied. Nowadays, the HBV infection has not been considered a contraindication for breastfeeding in the infant; therefore, the WHO has recommended that all infants born to infected mothers should be breastfed for at least 4 months and ideally 6 months.

## 5. Risk Factors for Vertical Transmission

**Figure 4:**
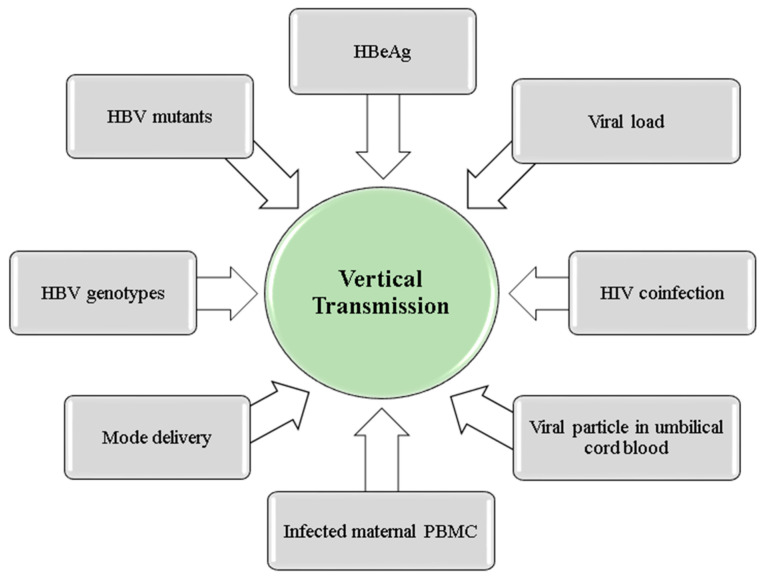
Factors associated with HBV vertical transmission.

Among the risk factors identified for mother-to-child HBV transmission are HBeAg, maternal viral load, coinfection with human immunodeficiency virus (HIV), viral genotype and HBV mutants, among others (Figure 4).

### 5.1. HBeAg

HBeAg is a secretory protein of 17 kDa. After transcription of the precore/core ORF, the precore protein is translated in a product that is processed in the reticulum endoplasmic prior to secretion. Although HBeAg is not required for viral replication, this antigen has a role on the establishment of chronic infection and on the development on immunotolerance [77,78]. 

The marker HBeAg is strongly associated with risk of HBV vertical transmission. Indeed, in a case-control study carried out in India, HBV transmission from mother to child was reported in 65% (13/20) of infants born to HBeAg-positive and HBV DNA-positive mothers [60]. While, only 9.1% (1/11) of babies were HBV infected in born to mothers negative for both the HBeAg and viral genome but positive for HBsAg. Similarly, a study in China demonstrated that 69.7% (23/33) of infants born from HBeAg-positive mothers were HBsAg positives at birth, while none of the infants born from HBeAg-negative mothers were positive [79]. In addition, mothers whose infants were infected with HBV had higher viral loads than mothers whose infants were not infected (*p* = 0.04). 

In fact, HBeAg is a marker of high-level viral replication and risk of vertical transmission. Furthermore, fetal exposure to HBeAg during pregnancy has been reported to lead to neonatal immunotolerance to HBV proteins and, consequently, to chronic infection in infants. This is explained by the ability of the viral protein to cross the placental barrier and generate tolerance of HBV-specific T cells and macrophages to the virus in the infant [80,81].

Animal models have been used to study the immunotolerance properties of HBeAg. Offspring were obtained by crossing female hemizygous HBV transgenic mice (TGD mice) to naive male mice; mouse pups exposed to maternal HBeAg were thus obtained [81]. Then, the offspring at 9 weeks were inoculated with the 1.3 mer HBV genome DNA into the liver, and HBV replication was demonstrated for 28 weeks in these animals. In contrast, inoculation of the HBV genome into mice born to HBV-negative females led to HBV elimination within 4 weeks. These results indicated that the intrauterine exposure to HBeAg establishes a persistent infection in the offspring. Interestingly, the population of INF-γ positive CD8+ T cells was 2.8% in the liver of control mice but only 0.5% in TGD mice. Additionally, CD8+ T cells in TGD mice expressed a high level of PD-1 (programmed death-1) and of PDL-1 (programmed death ligand-1) in Kupffer cells when compared with the control mice [82,83]. These interesting results suggest that HBV tolerance in the offspring of HBV-infected mothers may be due to decreased CD8 T cell responses to HBV that result from up regulation of PD-1 expression on CD8+ T cells and PD-L1 in Kupffer cells. Even though the ability of HBeAg to cross the murine placenta may not be enough evidence to prove that this also occurs in human placenta, it provided a possible mechanism to explain how the exposure to intrauterine HBeAg induces viral persistence and might favor vertical transmission. 

### 5.2. Maternal DNA Viral Load

Viral load has been identified as an important predictor and independent risk factor for HBV mother-to-child transmission. The prophylaxis effective rate of passive–active immunoprophylaxis is approximately 100% if viral load in the mother is <5.5 log 10 copies/mL before the delivery [84,85]. According to the results obtained by Singh et al. and Wiseman et al., a maternal viral load of >8 log 10 IU/mL is associated with failure of immunoprophylaxis, thus increasing the risk of mother-to-child transmission [60,86]. Moreover, a cohort study showed that infants born to HBeAg-positive mothers with viral load equal to or greater than 10^6^ copies/mL failed immunoprophylaxis [8]. Indeed, after stratification of pre-delivery viral loads in the mothers to <10^6^, 10^6^–10^7^, 10^7^–10^8^ and >10^8^ copies/mL, the rates of immunoprophylaxis failure were 0%, 3.2% (3/95), 6.7% (19/282) and 7.6% (5/66), respectively (*p* < 0.001). These findings suggest that viral load in mothers may have a significant impact on immunoprophylaxis failure. The current clinical practice and clinical trials are adopting the HBV DNA level above 10^8^ copies/mL as the threshold for intervention.

### 5.3. Human Immunodeficiency Virus Coinfection

Coinfection with HIV has been also identified as a risk factor of HBV vertical transmission [87,88]. A study in South Africa reported a rate of HBV vertical transmission of 28% in HIV/HBV co-infected pregnant women; they were HBeAg positive with an average viral load of 8.3 IU/mL before delivery [89]. In contrast, in a study in Malawi, infants born to HBeAg-positive women with a viral load of >7.5 log10 IU/mL had an HBV transmission rate of 10% [88]. The mothers of these two studies never received antiretroviral treatment, thus increasing the risk for both HIV and HBV transmission. It is important to note that the risk of vertical transmission could be due to the presence of HBeAg and high viral loads in mothers (10^7^–10^8^ copies/mL).

### 5.4. HBV Genotypes 

HBV is classified into ten main genotypes (A–J) and several subgenotypes [5,89,90].

Despite a similarly high prevalence of HBV chronic carriers, the vertical transmission rate in East Asia, particularly in China (10–88%), is higher than in sub-Saharan Africa (≤8%) [91,92,93]. In East Asia, where genotypes B and C are prevalent, most infected women of gestational age are HBeAg+ with high viral load [94]. Meanwhile, in sub-Saharan Africa where HBV genotypes A1 or E are prevalent, seroconversion to anti-HBe occurs before age 15 years old; therefore, most women of gestational age are anti-HBe [95]. However, other studies are not conclusive about the association between the HBV genotype and the risk of vertical transmission [96,97]. Further studies that include other genotypes, such as E, F, G and H, are necessary to define the role of HBV genotypes in mother-to-infant HBV transmission.

### 5.5. HBV Mutants 

The escape variants are non-synonymous mutations in the sequence encoding the “a” determinant of HBsAg. This is a highly conserved region overlapping with the region coding for viral polymerase, and is the major target for neutralizing antibodies against HBV. These mutations have been identified in individuals with HBV infection despite the presence of neutralizing antibodies.

In a study carried out in China, 41 mothers whose children were HBsAg negative and anti-HBs positive were compared with 37 mothers whose children were HBsAg positive 1 year after HBV vaccination [96]. Interestingly, T123A and G145R mutations were observed only in failure-group mothers; no significant difference in the total mutation rate was observed between the groups. These results suggested that a high viral load and specific mutations, such as T123A and G145R, could be an important risk factors for immunization failure. However, another study described different mutation sites in the HBV pre-S/S ORF including 105 silent mutations and 5 missense mutations (A826G, C531T, T667C, C512T and C546A), but without significant differences in mutation frequencies and the risk of vertical transmission [98]. More robust association studies are needed to confirm whether escape mutants could play an important role in vertical transmission.

Furthermore, A study on the presence of mutations in the precore region in sera from two infants with neonatal fulminant hepatitis B and their anti-HBe-positive mothers demonstrated the G28A mutation, which generates a stop codon in 18 HBV clones obtained from one of the mothers and in 31 clones from her infant [99]. Besides the stop codon G1896A has been described in cases of fulminant acute hepatitis B in young infants, but not in mothers. [100]. This mutation has also been observed in other studies [101,102]. These findings indicate that HBV mutants defective in the precore region in some carrier mothers are associated with fulminant hepatitis B in their babies.

In a case-control study carried out in Thailand, serum samples from 14 infected infants (13 HBeAg-positive and one HBeAg-negative) who had been previously vaccinated and their respective mothers were analyzed for HBV DNA, genotypes and S and precore/core variants [103]. Additionally, 30 serum samples from HBsAg-positive mothers (15 HBeAg positive and 15 HBeAg negative) whose infants were not infected and received the complete vaccination schedule served as control. The study showed that HBeAg-positive mothers had viral DNA titers equal to or higher than those of HBeAg-negative mothers. The infants and their respective mothers had the same HBeAg status and were infected by the same HBV genotypes (B or C). Furthermore, although D144G and G145K mutations were reported in two infants, these were not found in their mothers; this result indicates that these mutations may originate or be selected under immune pressure during the infection of the infants. On the other hand, PC mutations were found in HBeAg-negative mothers, and the A1762T/G1764A double mutation was reported in two infants born to HBeAg-positive mothers; however, these mutations were not found in their mothers. Interestingly, among HBeAg-positive mothers with uninfected infants, the presence of BCP mutations/deletions was more common than in HBeAg-positive mothers whose infants were infected [103]. 

These results suggest that BCP mutations/deletions could act as a protective factor in the mother-to-child transmission of HBV. Although the possible mechanism by which precore/BCP mutations may protect against vertical transmission of HBV has not yet been well studied, it is possible that down regulation of precore mRNA transcription and subsequent decreased HBeAg production caused by the double mutation in BCP may contribute to decreased immunotolerance and enhanced host immune response, resulting in viral clearance after possible perinatal transmission. 

## 6. Prevention of Vertical Transmission

The prevention of vertical transmission is an essential step in reducing the global prevalence of chronic HBV, mainly in endemic areas where HBV infections occur mostly during infancy and early childhood. The key to eliminating the vertical transmission is screening and preventive measures both during pregnancy and after delivery. In the screening, all pregnant women should be tested for HBsAg; if the results are positive, the viral DNA must be quantified and the status of the HBeAg determined. This information is important because it indicates the risk of vertical transmission and can be established as proper clinical management.

Currently two preventive measures have been established to avoid the vertical transmission of HBV: (1) immunoprophylaxis in child; (2) antiviral treatment for mother.

### 6.1. Immunoprophylaxis

Immunoprophylaxis includes the administration of HBIG and HBV vaccine. The HBIG is commonly administered to the newborn within 24 h of birth, as recommended by the Centers for Disease Control and Prevention. The HBIG provides immediate but temporary passive immunization [38,104]. A meta-analysis showed that HBIG significantly reduced the HBV vertical transmission rate (OR 0.5, CI 0.41–0.6) as compared to the placebo when given to the newborn at birth [105]. Although the HBIG prophylaxis can reduce HBV infection in the neonate, the standard immunoprophylaxis regimen consists of administration of both HBIG and HBV vaccine simultaneously at two different sites within 24 h of delivery. The birth dose should be followed by two (1–2 months and 6–8 months of age) or three doses (2, 4 and 6 months of age) with a minimum interval of four weeks. The dosages and schedules may vary according to the national routine immunization programs established in each country [1,106].

Some studies have shown that vaccination alone may be equally effective [107,108]. However, it has been demonstrated that the immunoprophylaxis using both HBV vaccine and HBIG reduce the vertical transmission from 90% to 5–10%. In the previously commented meta-analysis, children who received the HBV vaccine alone had a relative risk infection of 0.28, while adding HBIG reduced the risk to 0.08 when compared with patients who received a placebo or no intervention [105]. These findings demonstrate that adherence to the HBV immunoprophylaxis regimen in the neonate (vaccine and HBIG) prevents transmission of infection from HBeAg-positive mothers to their infants. However, immunoprophylaxis has some limitations. It is only effective against infection acquired via perinatal or postnatal transmission, and it does not protect against transplacental transmission [11,109,110]. The above-mentioned transplacental leakage or HBV-infected villous endothelial cells and infected maternal peripheral blood mononuclear cells are the possible mechanisms that explain the low protection of immunoprophylaxis in intrauterine transmission. Indeed, HBIG given to mothers in the third trimester has not been shown to be effective in reducing vertical transmission. A randomized trial of case-controls in China studying 250 HBeAg positive pregnant women (117 cases and 133 controls) and their neonates showed that there was no difference in the number of infected infants born to mothers who received three doses of HBIG in the third trimester of the pregnancy and those who did not. In addition, a high viral load and HBeAg positivity may increase the risk of immunoprophylaxis failure [109]. 

Another putative mechanism of failed immunoprophylaxis is escape mutants. These mutants are variants that are found in lower proportion in the viral population and are selected by immune pressure during HBV immunoprophylaxis; therefore, these strains could contribute to immunoprophylaxis failure. Although the impact that these mutations may have on the success of immunization programs has not been well elucidated, it is possible that these variants could infect previously immunized individuals. 

The G145R mutant located in the “a” determinant region of HBsAg is known to be the most important escape variant. Studies conducted in Taiwan to evaluate the efficacy of the vaccine and the occurrence of escape mutants have shown that the G145R mutation is the most frequent in the population since the introduction of the vaccine more than 20 years ago [111]. Similarly, the G145R mutation has been reported in a study conducted in children born to HBV-infected mothers who had the G145R mutant as a minority variant [108]. The study reported a G145R mutant frequency of 24% (6/25) in children who became infected due to the failure of immunoprophylaxis and of 10.3% (13/126) in children who had not received immunoprophylaxis. However,, the infection did not progress in any of the infants born to mothers infected with the G145R variant, despite that the mothers were HBeAg positive with high viral load [112]. 

Escape mutants have also been identified circulating in indigenous children and mothers in the state of Amazonas, in southeast Colombia [25]. The viral genome (S ORF) was detected in 8.3% (2/31) of serum samples obtained from anti-HBc positive children and in 3.1% (5/159) of samples from anti-HBc positive mothers. Four mutants were identified in three serum samples obtained from mothers, G145R and the two potential escape mutants L109R and G130E. Interestingly, the escape mutant W156* was identified in a sample obtained from a child having undergone the complete vaccination schedule; this mutation generates a stop codon resulting a truncated protein. Although, in this study the mutants were not detected in mother–child pairs, possibly because of minority sequences, it cannot be ruled out that they are not being transmitted from mother to child in these populations. Additional studies are necessary to elucidate the frequency and the epidemiological impact that these mutants have in vertical transmission and in the effectiveness of current vaccination strategies.

### 6.2. Antiviral Treatments

Some studies evaluated the efficacy of antiviral treatments in reducing the HBV vertical transmission rate [113,114,115,116,117,118,119]. A placebo-controlled study evaluated the efficacy of lamivudine in mothers with a high viral load (>1000 mEq/mL). A dose of 100 mg of lamivudine or placebo was administered from week 32 of pregnancy to week 4 postpartum to 150 mothers (149 were HBeAg-positive). The HBsAg positivity rate in infants was 18% in the lamivudine group and 39% in the placebo group [113]. The results of these studies suggest a reduction in HBV transmission in children born to mothers treated with lamivudine. Moreover, an observational study demonstrated that although treatment with lamivudine in the third trimester of pregnancy reduces viral load in women with antiviral treatment when compared with untreated women, the presence of mutants resistant to lamivudine (substitution in codons 181 and 204) can affect the response to treatment [114]. This study showed that lamivudine could have a poor antiviral effect in the third trimester, and it may generate resistant viral variants in a short time.

Given the limitations of lamivudine treatment, several trials demonstrated that Telbivudine was more effective at reducing maternal viral load. In a case-control study conducted in China, the antiviral effect of telbivudine was evaluated in 135 HBeAg-positive pregnant women with viral loads of >10^7^ copies/mL) [115]. Telbivudine treatment was started between 20 and 32 weeks of gestation and continued until 4 weeks postpartum or 28 weeks, depending on the alanine aminotransferase level. Telbivudine treatment was started between 20 and 32 weeks of gestation and continued until 4 weeks postpartum or 28 weeks, depending on the alanine aminotransferase level. In mothers who received the treatment, the viral load was reduced by an average of 2.44 ± 1.79 log10 copies/mL before delivery; the HBV genome was not detected in 33% of mothers when compared with the untreated controls. It is important to note that all babies in the study received combined prophylaxis with the HBIG/vaccine at birth. On the other hand, the HBsAg was not detected in infants born to telbivudine-treated mothers, while HBsAg positivity was 8% in infants of untreated mothers. It is important to mention that all the babies in the study received combined prophylaxis with HBIG/vaccine and none of them had birth defects in either group. The efficacy and safety of Telbivudine was also evaluated in a meta-analysis of six studies [116]. In this meta-analysis, the treatment group consisted of 306 mothers who received telbivudine in the second or third trimester of pregnancy until delivery or 1 month postpartum and a control group (270 mothers) who did not receive any antiviral treatment. Analyzes reported significantly lower HBsAg and viral genome positivity in newborns born to mothers in the telbivudine-treated group when compared to the control group; the newborns received the full regimen of immunoprophylaxis at birth. These results demonstrate that the administration of telbivudine at the end of pregnancy decreases the risk of intrauterine HBV infection without apparent adverse effects.

A third antiviral Tenofovir has been shown to be effective in reducing maternal viral load. The efficacy of this antiviral was evaluated in a retrospective study conducted in the third trimester of pregnancy in 11 women with viral loads of >10^6^ copies/mL. Interestingly, the maternal viral load decreased from 8.87 log 10 copies/mL to 5.25 log 10 copies/mL at delivery; none of the newborns were HBsAg positive and received passive/active prophylaxis. There were no complications during delivery or birth defects [117]. Similar results were obtained in a case-control study that included 45 HBeAg-positive pregnant women with high viral loads greater than 10^7^ copies/mL, 21 of them treated with tenofovir between weeks 18–27 of gestation and 24 untreated. There was no HBsAg positivity in infants born to tenofovir-treated mothers, but it was detected in infants of untreated mothers (8.3%). All infants received the full regimen of HBV immunoprophylaxis [118]. These findings suggest that tenofovir is safe and effective for the prevention of mother-to-child transmission of HBV.

The global recommendation is to use Tenofovir, Lamivudine and Telbivudine during pregnancy, but not Entecavir due to its questionable use. One study using a mice model demonstrates that the use of Entecavir could permeate the placental barrier and interfere with fetal development [119]. The study demonstrates that small amounts of ETV can cross the placenta in this animal model, which would indicate low fetal exposure to ETV. Finally, the use of other drugs such as interferon alpha and pegylated interferon is not indicated for vertical transmission prevention because they are not as effective in viral elimination and have many side effects on the patient.

Some experts recommend continued antiviral therapy after delivery, but its implications during breastfeeding are still unknown. A study of pregnant women treated with Lamivudine showed that breast milk contained only 684 ng/mL of Lamivudine in contrast to its blood level of 1070 ng/mL [120]. No correlation was found between HBV DNA detection in breast milk and child infection. Therefore, these findings support the clinical-based recommendations of the use of post-partum antiviral therapy in HBV positive women to eliminate this residual transmission.

## 7. Conclusions

HBV transmission from mother to infant still represents a major proportion of chronic hepatitis B cases in some regions of the world. Prevention of these infections is the key to controlling and finally eradicating HBV, fulfilling the WHO 2030 goal (an HBsAg prevalence in children of no more than 0.1%). Unfortunately, not all countries have 90% vaccination coverage and not all babies born to infected mothers receive immunoprophylaxis on time and the complete scheme. Additionally, both HBeAg positivity and high levels of viremia in mothers are factors that confer an additional risk for immunoprophylaxis failure. Therefore, it is necessary to identify HBsAg positive pregnant women and to provide optimum prophylaxis to their newborn children (vaccine combined with HBIG, with maternal antiviral drug treatment or all the three) principally in endemic areas.

## Figures and Tables

**Table 1:** Serological and molecular markers of HBV infection.

Markers	Clinical Significance
HBsAg	First serologic marker of infection; detectable 3 weeks before the onset of symptoms. The positivity of this marker for ≥6 months indicates chronicity.
HBeAg	Detectable from 3 to 6 weeks before development of symptoms; indicates high level of viral replication and is related to immune tolerance.
Anti-HBs	Detectable from 1 to 3 months after vaccination (antibody titre >10 mlU/mL) or after clinical resolution of acute infection. These two states can be distinguished by detection of anti-HBc-Total, which is present in subjects who had HBV infection but absent in vaccinated.
IgM anti-HBc	Marker of acute infection or recent infection; appears with the onset of symptoms and persists for up to 32 weeks after infection. Exceptionally, it can reappear during a flare in chronic hepatitis B.
Total anti-HBc	Marker of previous or ongoing infection with HBV; persists for life.
Anti-HBe	Marker of resolution if it is detected together with anti-HBs. Marker present in phases 3 and 4 of chronic infection.
HBV DNA	Viral replication (IU/mL).

Hepatitis B surface Antigen (HBsAg); Hepatitis B e Antigen (HBeAg); Antibody to Hepatitis B surface Antigen (Anti-HBs); Antibody to Hepatitis B e Antigen (Anti-HBe); Total antibody to hepatitis B core antigen (Total anti-HBc); IgM antibody to hepatitis B core antigen (IgM anti-HBc).

## Data Availability

Not applicable.
